# Pancreatic β-Cell Membrane Fluidity and Toxicity Induced by Human Islet Amyloid Polypeptide Species

**DOI:** 10.1038/srep21274

**Published:** 2016-02-16

**Authors:** Emily H. Pilkington, Esteban N. Gurzov, Aleksandr Kakinen, Sara A. Litwak, William J. Stanley, Thomas P. Davis, Pu Chun Ke

**Affiliations:** 1 ARC Centre of Excellence in Convergent Bio-Nano Science and Technology, Monash Institute of Pharmaceutical Sciences, Monash University, 381 Royal Parade, Parkville, VIC 3052, Australia; 2 St Vincent’s Institute of Medical Research, 9 Princes Street, Fitzroy, VIC 3065, Australia; 3 Department of Medicine, St. Vincent’s Hospital, The University of Melbourne, Melbourne, Australia; 4 Department of Chemistry, University of Warwick, Gibbet Hill, Coventry, CV4 7AL, United Kingdom

## Abstract

Aggregation of human islet amyloid polypeptide (hIAPP) into fibrils and plaques is associated with pancreatic β-cell loss in type 2 diabetes (T2D). However, due to the rapidness of hIAPP conversion in aqueous phase, exactly which hIAPP species is responsible for the observed toxicity and through what mechanisms remains ambiguous. In light of the importance of understanding hIAPP toxicity for T2D here we show a biophysical scheme based on the use of a lipophilic Laurdan dye for examining MIN6 cell membranes upon exposure to fresh and oligomeric hIAPP as well as mature amyloid. It has been found that all three hIAPP species, especially fresh hIAPP, enhanced membrane fluidity and caused losses in cell viability. The cell generation of reactive oxygen species (ROS), however, was the most pronounced with mature amyloid hIAPP. The correlation between changes in membrane fluidity and cell viability and their lack of correlation with ROS production suggest hIAPP toxicity is elicited through both physical and biochemical means. This study offers a new insight into β-cell toxicity induced by controlled hIAPP species, as well as new biophysical methodologies that may prove beneficial for the studies of T2D as well as neurological disorders.

Type 2 diabetes mellitus (T2D) is a metabolic disease currently affecting 9% of the global adult population^1^, with prevalence expected to double by 2035^2^. Though the major burden of the disease is largely preventable through healthy diet and exercise, T2D is predicted to become the seventh leading cause of death by 2030^3^. A key hallmark during the onset of T2D is dysfunction and death of pancreatic β-cells, located within the islets of Langerhans^4^,^5^. Once β-cell mass decreases by 40–60%, development of T2D is irreversible^4^. There is growing evidence that the 37-residue peptide human islet amyloid polypeptide (hIAPP), also known as amylin, directly contributes to β-cell loss^6^,^7^,^8^,^9^,^10^,^11^,^12^, and subsequently there is a need to establish the fundamental mechanisms of hIAPP-mediated toxicity.

hIAPP is co-secreted with insulin by pancreatic β-cells and largely contributes to glycemic control^13^. It is highly amyloidogenic, and aggregates kinetically in a concentration-dependent manner to form insoluble plaques and fibrils that are present in 90% of T2D patients^14^. The presence of β-cell granule components, including insulin at a 1–2:50 molar ratio to hIAPP, prevents aggregation of the peptide at high concentrations within healthy β-cells^15^,^16^,^17^. Consequently, a deviation in hIAPP secretion within a single cell can be capable of initiating amyloid fibrillation^18^, and recent evidence has also suggested that amyloid could act to trigger amyloidosis in monomeric hIAPP in a prion-like mechanism^19^. It has therefore been hypothesised that intracellular amyloidosis can trigger death of the β-cell and provide a ‘seed’ for larger plaque formation extracellularly^18^. However, there has been considerable debate in the literature as to the primary hIAPP conformation that induces β-cell toxicity^20^–namely, monomeric, oligomeric, growing fibrils (also referred to as protofibrils) or mature amyloid hIAPP–and, additionally, the mechanisms thereof. Within an aqueous environment, hIAPP fibrillates rapidly, and multiple forms can co-exist at any given stage^21^. When compared to the well-characterised amyloid-β polypeptide (Aβ) implicated in Alzheimer’s disease, which has a far slower aggregation rate of hours to days, isolation of different hIAPP species to examine their cytotoxic effects has thus far been difficult to accomplish^20^, further contributing to ambiguity with regards to hIAPP toxicity.

Early research of the past two decades favoured amyloid as a causative agent of β-cell failure, thought to be mediated through physical association between the plaques and the cells, leading to membrane perturbation, production of reactive oxygen species (ROS), and/or apoptosis^22^. Clinical studies in a Japanese population who produced a mutated form of hIAPP with an increased aggregation propensity showed that they subsequently developed T2D^23^,^24^. Lorenzo *et al.* proposed that β-cell viability is only reduced when hIAPP concentration is high enough to mediate fibrillation, and identified amyloid-membrane contact triggering apoptosis as the primary mechanism of toxicity^8^. Schubert *et al.* screened a number of amyloid peptides in PC12 and B12 cells for production of ROS with 2,7-dichlorofluorescin diacetate, and determined that hIAPP was correlated with ROS production and subsequent loss of cell viability, while no such species were measured with the non-amyloidogenic rat-derived IAPP^12^. There is evidence that the hydrophobic amyloid can mediate formation of ion channels or pores in the β-cell membrane, through its high propensity for contacting and integrating phospholipids into growing fibrils, leading to cell death by unregulated calcium ion influx or cytosol leakage^25^,^26^,^27^,^28^.

In more recent years, however, the focus has shifted to the soluble oligomeric form of hIAPP as the main toxic species, but considering similar mechanisms of toxicity as were postulated for hIAPP amyloids. In light of the biophysical and biochemical connections between hIAPP and Aβ fibrillation, the working paradigms concerning hIAPP toxicity have been influenced by the mechanistic studies of Alzheimer’s disease, an approach which remains to be validated. Ritzel *et al.* observed that hIAPP oligomers mediated a disruption in islet architecture *ex vivo*, impairing cell coupling and insulin secretion and inducing apoptosis^11^. Research from Kayed *et al.* and Meier *et al.* described cell membrane permeabilisation induced by hIAPP oligomers, while monomeric hIAPP or amyloid fibrils showed no adverse effects^9^, and prevention of amyloid fibrillation did not mitigate toxicity^10^. The latter study utilised rifampicin to prevent the formation of amyloid plaques, demonstrating its capacity to prevent fibrillation through Thioflavin-T (ThT) staining–an established method to detect amyloid protein. However, research by Meng *et al.* refuted this, revealing that rifampicin had no effect on hIAPP fibrillation and demonstrating that the ligand can interfere with ThT fluorescence^29^; thus illustrating limitations of current methodology to characterise hIAPP species, and proving that the exact science of hIAPP-mediated toxicity is still unclear.

In the present study, we sought to further investigate fresh and oligomeric hIAPP, as well as mature amyloid as toxic agents to pancreatic β-cells (Fig. 1A), and attempted to establish their respective mechanisms of action. We additionally explored new methodologies to characterise and control hIAPP toxicity and fibrillation, respectively. Resveratrol, a polyphenol derived from red wine, proved a suitable candidate for prevention of amyloid fibrillation^30^,^31^,^32^, and was employed in the current study to render hIAPP oligomers through off-pathway molecular self-assembly^30^. Ratiometric imaging, a dual-channel confocal fluorescence technique, was used to visualise cell membrane perturbation by each hIAPP species (Fig. 1A,B). This was achieved via the employment of a lipophilic Laurdan dye, utilised as a probe for membrane lipid order^33^,^34^,^35^,^36^ and applied to a number of applications, including nanoparticle exocytosis^37^, viral budding^38^, and yeast reproduction^39^. Cell uptake and intracellular effects of the peptide were not a focus of this present study due to the complexity of labelling the hIAPP species without altering their conformations. The presence of ROS was also investigated to determine any correlation between membrane perturbation, ROS production and β-cell viability upon hIAPP exposure. We propose that oligomeric hIAPP, growing fibrils and mature amyloid are, though to different extents, toxic to pancreatic β-cells, but their modes of toxicity are not necessarily conserved between each hIAPP species. In addition to its direct implication for research in T2D, this study demonstrates the use of ratiometric imaging as an effective tool for examining the biophysical and toxicological manifestations of hIAPP that remain a challenge due to the kinetic nature of this most aggregation prone polypeptide.

This paper is arranged as follows. We first present a characterisation of hIAPP in aqueous and at micromolar concentrations, rendering in three states as fresh and oligomeric hIAPP (stabilised by resveratrol) as well as mature amyloids, using high-throughput dynamic light scattering (DLS). We then show our ratiometric imaging of MIN6 pancreatic cells resulting from their exposure to hIAPP of the three states, quantified by generalised polarisation (GP) of the cell membranes partitioned with a Laurdan dye. Finally we compare our GP measurement with the assays of MIN6 cell viability and ROS generation induced by hIAPP of the three states and draw conclusions of the present study. Implications of understanding protein aggregation for research in neurological disorders and T2D are discussed at the end.

## Results

### hIAPP of three states

High-throughput DLS was utilised to observe hIAPP amyloid fibrillation in aqueous solution. In order to investigate the specific properties of oligomeric hIAPP, polyphenol resveratrol was utilised as a fibrillation inhibitor. As shown in Fig. 2A, freshly dissolved monomeric hIAPP (approximately 2~3 nm) readily formed large amyloid fibrils within several hours (hydrodynamic radii of 0.1~4 μm). When fresh hIAPP was incubated with resveratrol at a 2:1 molar ratio, the hydrodynamic radius of hIAPP remained consistent at 3~5 nm over 7.5 h, demonstrating that peptide fibrillation was effectively inhibited and stabilised by the polyphenol. Considering the established dynamic process of hIAPP fibrillation, including nucleation, elongation and saturation of amyloid formation, we term the peptides immediately dispersed in water as “fresh”, stabilised by resveratrol as “oligomeric”, and over 2 weeks as “mature”. Based on the DLS measurement, the “fresh” samples consisted initially of hIAPP monomers but evolved into oligomers and fibrils over a timescale of several hours^40^ and the “oligomeric” samples were stable supramolecular assemblies of hIAPP-resveratrol formed on the spatial scale of a few nanometers over the timescale of nanoseconds (discrete molecular dynamics simulations, unpublished data). The “mature” amyloid samples, as visualised by TEM in Fig. 2B, were characterised by hIAPP amyloid fibrils of around 5–15 nm in diameter and tens of nanometers to micrometers in length^41^ and mostly devoid of monomers and oligomers.

### Membrane disorders induced by hIAPP of three states

**Figure 1:**
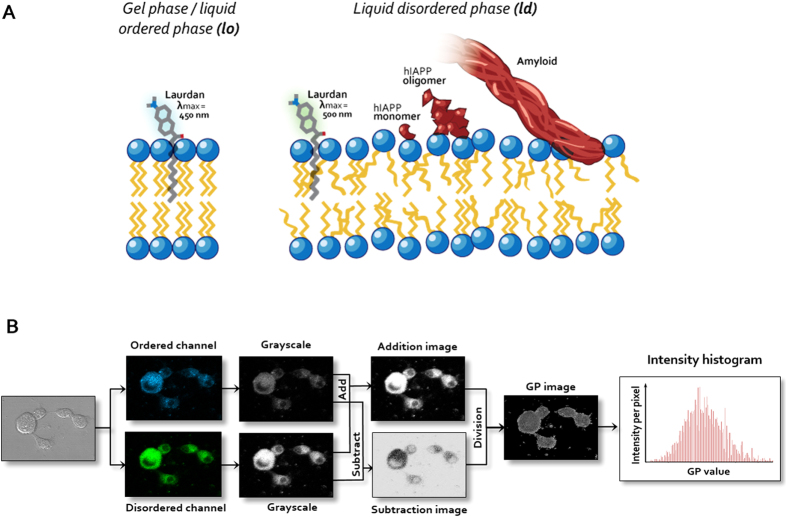
Laurdan dye as an indicator of cell membrane lipid order and ratiometric imaging. Laurdan (6-Dodecanoyl-2-dimethylaminonaphthalene) is a lipophilic dye capable of partitioning into cell phospholipid membranes (**A**). When excited at the 405 nm laser line, Laurdan emits fluorescence at 450 nm when the cell membrane is in the gel/liquid ordered phase (*l*_*o*_; left panel), and redshifts to 500 nm when the cell membrane is in the liquid disordered phase (*l*_*d*_; right panel). hIAPP monomers, oligomers and amyloid fibrils are predicted to cause lipid disorder (**A**). Intensity shifts between the ordered and disordered channels can be quantified as generalised polarisation (GP) values. A flowchart outlining the calculation of GP values from raw ratiometric confocal images in the ordered and disordered channels is represented by (**B**).

**Figure 2:**
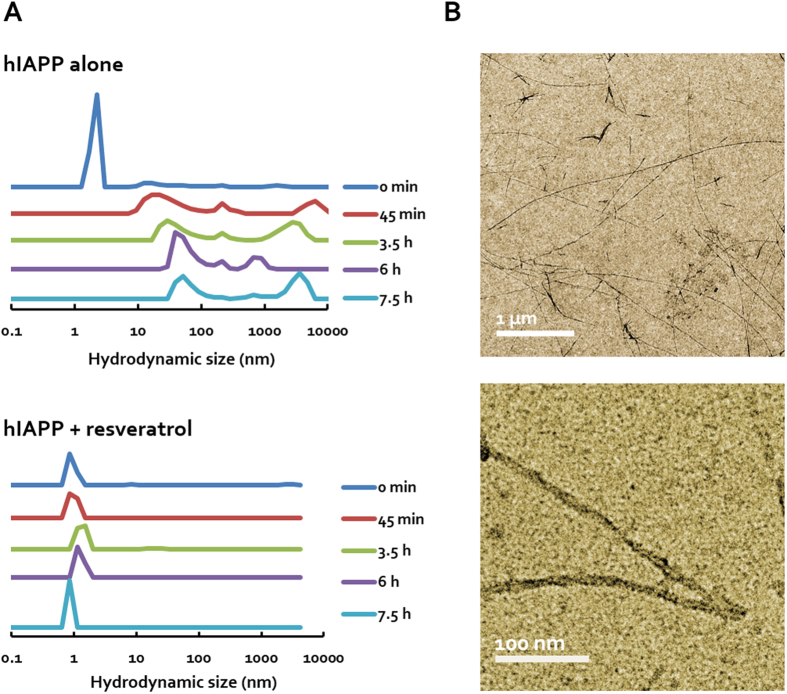
Characterisation of hIAPP species. (**A**) Dynamic light scattering shows that monomeric hIAPP (0 min) readily aggregated to form large fibrils in aqueous solution over time (450 min), but was stabilised in oligomeric form by resveratrol. (**B**) TEM images of mature amyloid fibrils and plaques (2 weeks old). The characteristic helical structure of the fibrils can be seen in the lower image.

**Figure 3:**
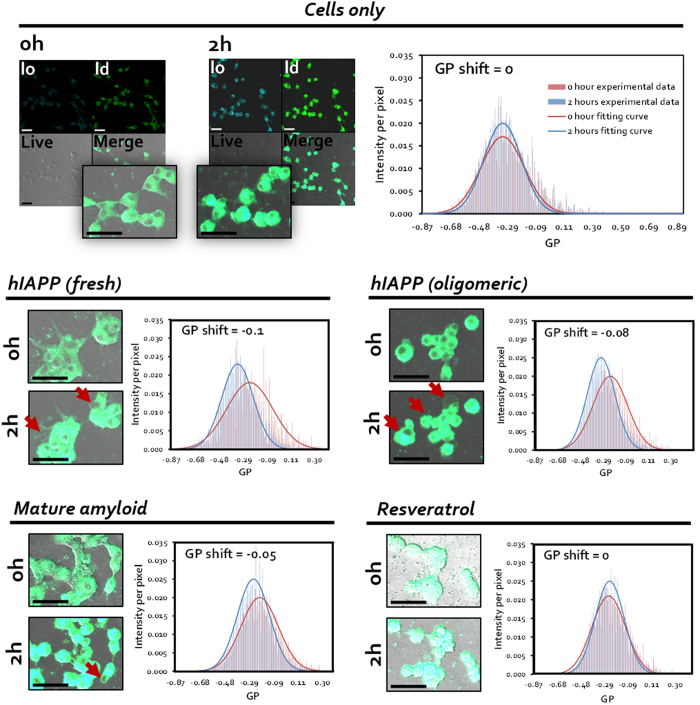
Ratiometric imaging of MIN6 cells treated for 2 h with fresh and stabilised oligomeric hIAPP, as well as mature hIAPP amyloid. The shift in GP values for MIN6 cells labelled with Laurdan dye was recorded over 2 h (B). Blue channel = *l*_*o*_ phase; green channel = *l*_*d*_ phase; greyscale channel = live/bright-field; blue-green channel = merge. Inset panels represent merged channels. Arrows indicate signs of unhealthy cells, including shrinkage, blebbing/cytoplasm leakage, and membrane disorder. Scale bars: 40 μm.

Given that a potential pathway of hIAPP-mediated cytotoxicity involves membrane perturbation, we investigated the interaction of fresh hIAPP, stabilised oligomeric hIAPP and preformed amyloid fibrils with the cell membrane of pancreatic β-cells using ratiometric imaging. Data was collected over 2 h to reduce interference from laser-mediated UV damage to cells. The lipophilic dye Laurdan (Fig. 1A) was utilised as a reporter for cell membrane lipid order, due to its capability to undergo spectral redshifting upon an increase in membrane fluidity^42^. Changes in cell membrane lipid order, namely between the gel/liquid ordered phase (*l*_*o*_, predominantly fluorescent at 430~470 nm) and the liquid disordered phase (*l*_*d*_, predominantly fluorescent at 480~550 nm), were determined by generalised polarisation (GP, see Fig. 1B & Methods). A negative GP shift, indicating decreased membrane lipid order, was observed in all cells treated with hIAPP species, while membrane lipid order did not change in control cells, or cells incubated with resveratrol only (Fig. 3, right panels). The largest GP shift occurred with the fresh, or fibrillating, hIAPP (−0.1), followed by oligomeric (−0.08) and amyloid (−0.05) species. Accordingly, at 2 h the morphology of the cells exposed to hIAPP indicates they are unhealthy (Fig. 3, left panels)–shrinkage and cytoplasm leakage/blebbing was observed in samples where the GP displays a net negative shift.

### Pancreatic β-cell viability upon exposure to hIAPP

**Figure 4:**
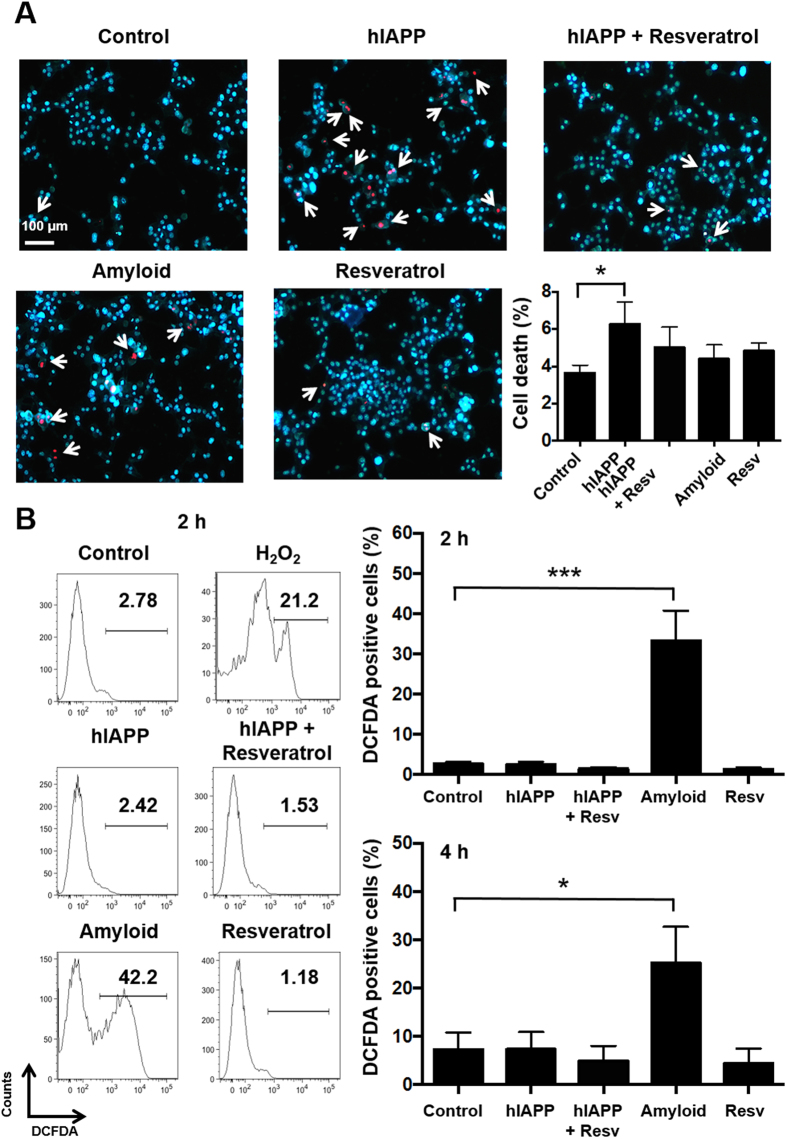
Viability and ROS production in MIN6 cells treated with fresh, stabilised oligomeric and mature amyloid hIAPP. MIN6 cells were left untreated (control) or incubated with fresh hIAPP, stable oligomeric hIAPP, mature amyloid or resveratrol alone. (**A**) Cell viability after 24 h of treatment was evaluated by Hoechst-33342 (blue) as a live stain, and propidium iodide (red) as a dead stain. White arrows indicate propidium iodide positive cells. Data shown are means and SEM of 3 independent experiments. *P < 0.05. Scale bar: 100 μm. (**B**) ROS positive cells were identified by DCFDA staining after treatment for 2 and 4 h. Representative FACS profiles after 2 h treatment are shown. H_2_O_2_ (1.96 mM) was used as positive control. Data shown are means and SEM of 4–6 independent experiments, *P < 0.05, ***P < 0.001.

Next, we determined what phase of hIAPP aggregation is responsible for toxicity to pancreatic β-cells. For this purpose, MIN6 cells were treated with fresh or aged aqueous hIAPP (10 μM) and/or resveratrol (20 μM) to obtain the three stages of the polypeptide. After 24 h treatment, cells were labelled with the DNA binding dyes Hoechst-33342 and propidium iodide to discriminate viable cells from dead cells. Hoechst-33342 labels dsDNA blue and is able to diffuse freely through intact and damaged membranes. Propidium iodide labels dsDNA as red, however due to its molecular properties, is impermeable to cells with intact membranes, thus staining only dead cells. Cell death was quantified by blue-red fluorescence or by fragmented nuclei, to the exclusion of viable cells identified by intact blue nuclei. We observed that treatment of MIN6 cells with fresh hIAPP for 24 h resulted in the greatest induction of cell death (6.2%, up from 3.7% of control) (Fig. 4A). Treatment with either the oligomeric form of hIAPP or mature amyloid also induced cell death to a minor extent (4.9% and 4.5%, respectively), while treatment with resveratrol alone also slightly affected cell viability (4.7%).

### ROS production in β-cells exposed to hIAPP

We then determined whether ROS production correlated with cell toxicity dependent on the aggregation state of hIAPP. To analyse this MIN6 cells were stained with 2,7-dichlorofluorescein diacetate (DCFDA) and subsequently treated with the three different forms of hIAPP. DCFDA is a fluorogenic dye that diffuses readily into the cell and is then deacetylated by cellular esterases to a non-fluorescent compound. After exposure to ROS, DCFDA is oxidised into 2,7-dichlorofluorescein (DCF). DCF fluorescence can be measured by flow cytometry through excitation at 488 nm, and is detectable at 535 nm. After 2 h and 4 h treatments with the three different forms of hIAPP we observed that treatment with mature amyloid fibrils resulted in the highest induction of ROS inside cells (up from 3% to 33% for 2 h treatment, and up from 7% to 26% for 4 h treatment, Fig. 4B). Treatment with fresh hIAPP had no effect on ROS production, while treatment with oligomeric hIAPP stabilised with resveratrol, or with resveratrol alone, tends to decrease ROS production in comparison to the control cells. Importantly, fresh and mature hIAPP had no effect on DCFDA emission (Supplementary Figure S1).

## Discussion

The premise of this study was to investigate the impact of different stages in the hIAPP fibrillation process on membrane fluidity, and the ways in which these different stages contribute to β-cell toxicity. Determining the fundamental means by which hIAPP mediates cell dysfunction and death is crucial not only for the prevention and management of T2D, but also for a variety of other health concerns, as hIAPP toxicity can present an issue to the body at large. Amyloid deposition has been observed to extend to the vascular system, heart, lungs and kidneys, and additionally hIAPP is capable of crossing the blood brain barrier–co-localisation with Aβ plaques in the central nervous system may cause increased morbidity in Alzheimer’s disease^43^,^44^.

Ratiometric imaging has proved to be a viable method for characterising membrane fluidity in the presence and absence of hIAPP. We observed good correlations between hIAPP-cell membrane interaction, as determined by GP values (Fig. 3), and decrease in β-cell viability (Fig. 3, confocal images; Fig. 4A) within 24 h after hIAPP exposure. In accordance with the loss in viability, observed in relation to a decrease in membrane lipid order mediated by these forms of hIAPP, and given the physical attributes of each hIAPP species, several hypotheses can be made for their modes of action. Structurally, oligomeric hIAPP–with or without the involvement of polyphenol resveratrol–could mediate H-H bonding with phospholipids to result in membrane disruption or damage. Mature amyloids can physically partition into the amphiphilic membrane, where the fatty acid tails bind to the fibrils through hydrophobic-hydrophobic interactions. In terms of fibrillation of hIAPP at the cell membrane, a two-step process for insertion has recently been proposed utilising model membranes. Monomeric hIAPP and early fibrils can partition into the membrane during the process of fibrillation as a result of their structural transformations and exposure of hydrophobic moieties, leading to changes in area per lipid molecule (and hence membrane fluidity) as well as release of calcium ions; further membrane damage is inhibited, but the ion release subsequently promotes fibril elongation at the membrane surface through incorporation of membrane lipids into growing fibrils^45^,^46^,^47^. This could provide an explanation for the ‘fresh’ hIAPP mediating the largest GP shift of the hIAPP species examined (Fig. 3). Overall, these data support published research^25^,^26^,^27^,^28^, but also illustrate the differential capacity of hIAPP of three states in physically disrupting β-cell membranes, which could lead to unregulated ion exchange and cytosol leakage, and subsequently cell death. Additionally, interactions between the hIAPP species and cell membranes, through H-bonding and/or hydrophobic interaction, could hinder the fibrillation process of the peptide via reduced availability of the peptide monomers and compromised cohesiveness of the peptide assembly architecture. Such reverse effect of hIAPP-membrane interaction on peptide fibrillation is an interesting new topic to be explored in future studies, as it could also elicit consequences on ROS production and β-cell viability.

Mechanisms of toxicity from other well-characterised amyloids, in particular Aβ, are frequently cross-referenced to characterise hIAPP toxicity. Aβ is proposed to have protective properties against ROS, one mode of action being as a metal ion clearance agent^48^, facilitated by three histidine residues^49^. Though hIAPP-copper(II) interactions do prevent amyloid fibrillation, any protective effect this provides against ROS generation and subsequent β-cell death is disputed^50^,^51^. It is known that β-cells are particularly vulnerable to ROS, and that it contributes to β-cell dysfunction during development of T2D^52^. In our study significantly elevated ROS production was associated with mature amyloid fibrils (Fig. 4B), but levels of ROS did not change from the control when β-cells were treated with fresh hIAPP. In the presence of resveratrol, ROS were reduced in comparison to the control cells, illustrating the excellent antioxidant properties of the polyphenol. The contrast between negligible ROS in growing fibrils generated from ‘fresh’ hIAPP after 2 h and 4 h and increased ROS production in mature amyloid suggests that properties of larger fibrils, including increased size and hydrophobicity, determined the scale of ROS production. Given these data, it is likely that the partitioning of amyloid into the cell membrane induces oxidative stress in the intracellular environment^53^, similarly to what has been observed in other fibrous hydrophobic materials, e.g. carbon nanotubes^54^,^55^. Consequent changes in the membrane fluidity could mediate further biochemical and biological processes–such as metal ion concentrations and transport, as well as metal ion association/chelation with the amyloid–and collectively result in β-cell death. It has additionally been demonstrated in literature that the disassembly of amyloid fibrils, mediated by polyphenols, generates ROS through a methionine-independent pathway^56^, which may need to be a consideration when designing constructs for the purpose of disassembling amyloid deposits. Mitigation of ROS does not prevent cell death in all hIAPP species, however, as evidenced by the data from cells treated with fresh and oligomeric hIAPP (Fig. 4). Subsequently, ROS production likely plays a significant part in hIAPP-mediated toxicity, but does not constitute the major mechanism of toxicity for the species considered.

In conclusion, we propose that oligomeric, fibrillating and amyloid hIAPP to different extents are all toxic to pancreatic β-cells, and that both membrane perturbation and ROS production contribute to hIAPP toxicity. Membrane perturbation, through H-bonding, hydrophobic interaction and physical penetration, represents an overarching mechanism of toxicity for each of the three respective hIAPP species examined. Amyloid hIAPP, additionally, induced marked ROS production in β-cells. Collectively, the observed correlation between changes in membrane fluidity and cell viability and their lack of correlation with ROS production suggest hIAPP toxicity is elicited through both physical and biochemical means. Lastly, this study has demonstrated new methodologies of ratiometric confocal fluorescence imaging and high-throughput DLS for characterising hIAPP species and their toxicity in β-cells, presenting biophysical means that have been recognised lacking^5^ in investigating hIAPP toxicity for T2D. Such methodologies, owing to their spatial and temporal resolution that are deemed sufficient for capturing peptide aggregation, should be extendable to the studies of other amyloidogenic disorders associated with ageing.

## Methods

### Materials

Human islet amyloid polypeptide (hIAPP, or amylin) (KCNTATCATQRLANFLVHSSNNFGAI-LSSTNVGSNTY; disulfide bridge: 2–7; MW: 3,906) was obtained from Abcam in lyophilised powder form. The hIAPP was weighed on a Cubis MSE balance (Sartorius, 0.01 mg resolution) and dissolved in Milli-Q water to form a 1 mg/mL (256 μM) stock solution immediately prior to (freshly-dissolved monomers) or around two weeks before (pre-formed/“mature” amyloid fibrils) commencing measurements. Resveratrol (purity > 99%; MW: 228) was obtained from Sigma Aldrich and dissolved in Milli-Q water to form a 120 μM (0.027 mg/mL) stock solution. Laurdan dye (6-Dodecanoyl-2-dimethylaminonaphthalene; MW: 354) was obtained from AnaSpec and maintained as a 4 mM stock solution (1.4 mg/mL) in DMSO, with a 500 μM solution in Milli-Q made up immediately prior to addition to wells. Hoechst-33342 was obtained from Sigma Aldrich and propidium iodide was obtained from Life Technologies, both were made to stock concentrations of 10 mg/mL in PBS. The DCFDA cellular ROS detection kit was purchased from Abcam.

### High-throughput dynamic light scattering

The hydrodynamic sizes of hIAPP and hIAPP-resveratrol were acquired at room temperature over 430 min using an automated, high-throughput DLS device (DynaPro Plate Reader, Wyatt; instrument resolution: 0.5 nm), with samples plated in quadruplicate in black 384-well plates (Thermo Fisher). Each well contained a total volume of 20 μl of either 10 μM hIAPP or resveratrol (20 μM) mixed with hIAPP at a 1:2 molar ratio in aqueous solution. To ensure good mixing, samples were spun for 1 min at 1,000 rpm/164 RCF (Centrifuge 5804, Eppendorf) prior to the start of the experiment. 20 data points for each sample well were collected by an optical module while scanning through all samples continuously. Processing of the acquired data was automated through the Dynamics 7.1.7 software.

### Transmission electron microscopy

Mature amyloid fibrils were visualised by TEM as follows: a 5 μl aliquot of amyloid hIAPP (25 μM in Milli-Q, ~2 weeks old) was pipetted onto a copper grid (300 mesh, carbon-coated) and allowed 60 s of adsorption. Excess sample was then drawn off using filter paper and the grids washed using 10 μL ddH_2_O, with excess drawn off as previously described. The grids were stained with 5 μL 1% uranyl acetate for 30 s, with excess stain drawn off. Grids were air-dried as needed. Electron microscopy was undertaken utilising a Tecnai G^2^ F30 Transmission Electron Microscope (FEI, Eindhoven, The Netherlands), operating at a voltage of 300 kV. Images were recorded using a Gatan UltraScan 1000 (2k × 2k) CCD camera (Gatan, California, USA) using Gatan Microscopy Suite control software.

### Ratiometric imaging

MIN6 pancreatic β-cells were seeded in 300 μL DMEM (Invitrogen, UK), containing 10% fetal calf serum onto 8-well slide plates (μ-Slide, Ibidi, Germany) coated with poly-D-lysine. Cells were allowed to attach overnight in a humidified, 37 °C, 5% CO_2_ incubator. Prior to imaging, old media was removed and replaced with serum-free Opti-MEM (Life Technologies), with cells washed in between using Opti-MEM. Laurdan dye was added to each well to a final concentration of 50 μM and allowed to equilibrate with the cell membranes for at least 30 min. Cells were transferred to a Leica SP8 inverted confocal fluorescence microscope housed in a humidified, 37 °C, 5% CO_2_ environment. Appropriate areas of each well, containing at least 10 viable cells, were identified. Freshly dissolved hIAPP monomers (10 μM), pre-formed amyloid fibrils (10 μM), resveratrol (20 μM), and fresh hIAPP monomers + resveratrol in a 1:2 molar ratio were added to respective wells. Imaging was undertaken at 1~30 min timepoints for 2 h with a 20 × /0.70 dry objective. Laurdan dye integrated into cell membranes was excited along the 405 nm laser line and emission read at 430~470 nm (representing the lipid membrane at the gel/liquid ordered phase) or 480~550 nm (representing the lipid membrane at the liquid disordered phase). To calibrate dye background levels, a well containing dye only was excited on the 405 nm laser line using 0.5 and 2 × laser power. An additional control of mixing individual hIAPP species with Laurdan abiotically did not show any noticeable effect on the dye fluorescence in aqueous solution (Supplementary Figure S2). Post-experimental 3D images were taken on a 63 × /1.40 oil immersion objective. Images were false-coloured and any adjustments made using LAS X software (Leica).

### Determination of generalised polarization (*GP*)

The acquisition of GP images was performed using the ImageJ (National Institute of Health) software and custom-written macro by Owen *et al.* (2011)^33^, with modifications. GP values were then calculated for each pixel of a cell membrane according to the following equation:


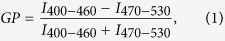


where *I* represents the intensity of pixels in the areas of interest in the image acquired in the ordered (430~470 nm) and disordered (480~550 nm) spectral channels. The sample pool for GP analysis was an average value of 25 cells (each cell 960 ± 330 pixels) from each image, totalling ~25,000 data points for each established histogram. GP shift was observed by subtraction of the GP distribution peak maximum of each sample with 2 h of incubation time from the GP value of the image taken at the beginning of the experiment (0 h).

### Cell culture and viability

The insulin-producing MIN6 cell line was cultured in DMEM (Invitrogen, UK) supplemented with 10% fetal calf serum. The percentage cell death of MIN6 was determined by labelling cells with the DNA dyes Hoechst-33342 (10 μg/mL) and propidium iodide (5 μg/mL) and counting at least 600 cells per experimental condition via inverted fluorescence microscopy. Cells were treated with 10 μM of fresh hIAPP, 10 μM of stabilised oligomeric hIAPP (with resveratrol 20 μM), 10 μM of preformed amyloid fibrils, or 20 μM of resveratrol for 24 h in Opti-MEM media (Life Technologies).

### Reactive Oxygen Species detection

ROS detection was performed using a DCFDA cellular ROS detection kit (Abcam). Mixing individual hIAPP species with DCFDA abiotically did not show any effect on the dye fluorescence in aqueous solution (Supplementary Figure S1). Single cell suspensions of MIN6 cells were stained with 200 nM of DCFDA for 30 min and subsequently treated with fresh hIAPP, stabilised oligomeric hIAPP and mature amyloid for 2 h and 4 h to avoid death of control cells in suspension. ROS levels were then measured indirectly by the oxidation of nonfluorescent DCFDA to fluorescent DCF by a flow cytometer, exciting the dye at 488 nm and detection at 535 nm. The percentage of ROS positive cells was then quantified using FlowJo Software, analysing the ROS^hi^ (DCF^+^) population.

### Statistical analysis

Data are represented as means ± SEM. Given the paired nature of the experimental design comparisons between treated groups were made by analysis of variance (ANOVA). A p value < 0.05 was considered statistically significant.

## Additional Information

**How to cite this article**: Pilkington, E. H. *et al.* Pancreatic β-Cell Membrane Fluidity and Toxicity Induced by Human Islet Amyloid Polypeptide Species. *Sci. Rep.* **6**, 21274; doi: 10.1038/srep21274 (2016).

## Supplementary Material

Supplementary Information
