# Supplementary material for: Pancreatic β-Cell Membrane Fluidity and Toxicity Induced by Human Islet Amyloid Polypeptide Species
Source: Sci Rep. 2016 Feb 16;6:21274. doi: 10.1038/srep21274 (PMC4754679; doi:10.1038/srep21274)
Supplement: Supplementary Information [file srep21274-s1.pdf]

Supporting Information

Pancreatic β-Cell Membrane Fluidity and Toxicity Induced by Human Islet Amyloid Polypeptide Species

Emily H. Pilkington,[1,5] Esteban N. Gurzov,[2,3,5] Aleksandr Kakinen,[1] Sara A. Litwak,[2] William J. Stanley,[2,3] Thomas P. Davis,[1,4] and Pu Chun Ke[1*]*

[1]ARC Centre of Excellence in Convergent Bio-Nano Science and Technology, Monash Institute of Pharmaceutical Sciences, Monash University, 381 Royal Parade, Parkville, VIC 3052, Australia

[2]St Vincent's Institute of Medical Research, 9 Princes Street, Fitzroy, VIC 3065, Australia

[3]Department of Medicine, St. Vincent's Hospital, The University of Melbourne, Melbourne, Australia

[4]Department of Chemistry, University of Warwick, Gibbet Hill, Coventry, CV4 7AL, United Kingdom

[5]These authors contributed equally to this work.

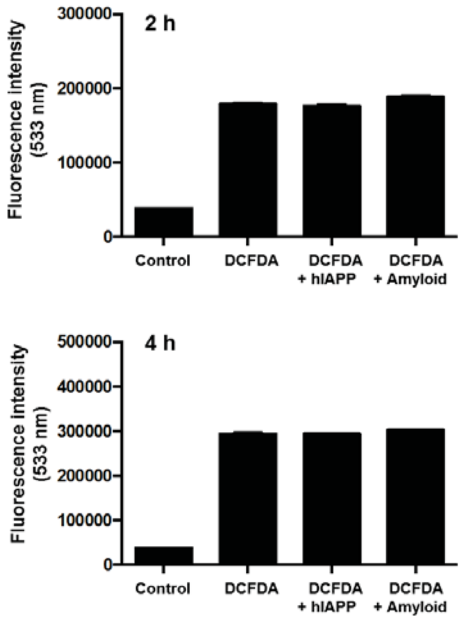

Figure S1. Fluorescence intensity measured after 2 h and 4 h exposure of DCFDA containing media to 10 μM fresh or amyloid hIAPP. The results are triplicates representative of two independent experiments.

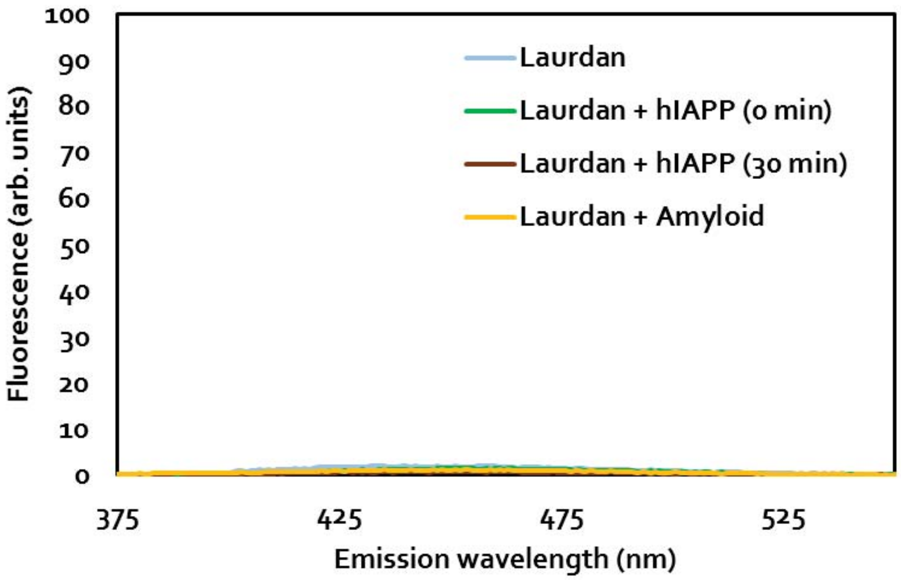

Figure S2. Fluorescence intensities of Laurdan dye in abiotic aqueous solutions, in the absence and presence of fresh hIAPP at 0 (i.e., monomers) and 30 min (i.e., monomers, oligomers and small fibrils) and amyloid hIAPP. The Laurdan emissions are negligible in all four cases due to the absence of lipid membranes. Concentration of hIAPP species: 10 μM. Concentration of Laurdan: 50 μM.
